# Top2a identifies and provides epigenetic rationale for novel combination therapeutic strategies for aggressive prostate cancer

**DOI:** 10.18632/oncotarget.3077

**Published:** 2014-12-26

**Authors:** Jason S. Kirk, Kevin Schaarschuch, Zafardjan Dalimov, Elena Lasorsa, ShengYu Ku, Swathi Ramakrishnan, Qiang Hu, Gissou Azabdaftari, Jianmin Wang, Roberto Pili, Leigh Ellis

**Affiliations:** ^1^ Genitourinary Program, Roswell Park Cancer Institute, Buffalo, NY; ^2^ Department of Medicine, University of Szeged, Hungary; ^3^ Department of Pharmacology and Therapeutics, Roswell Park Cancer Institute, Buffalo, NY; ^4^ Department of Cancer Prevention and Pathology, Roswell Park Cancer Institute, Buffalo, NY; ^5^ Department of Bioinformatics and Statistics, Roswell Park Cancer Institute, Buffalo, NY; ^6^ Department of Pathology, Roswell Park Cancer Institute, Buffalo, NY; ^7^ Department of Medicine, Roswell Park Cancer Institute, Buffalo, NY

**Keywords:** Top2a, etoposide, epigenetics, Ezh2, prostate cancer, therapy

## Abstract

Progression of aggressive prostate cancers (PCa) with androgen receptor splice variants or neuroendrocrine features is currently untreatable in the clinic. Therefore novel therapies are urgently required. We conducted RNA-seq using tumors from a unique murine transplant mouse model which spontaneously progresses to metastatic disease. Differential gene expression analysis revealed a significant increase of topoisomerase IIα, *Top2a* (Top2a) in metastatic tumors. Interrogation of human data revealed that increased Top2a expression in primary tumors selected patients with more aggressive disease. Further, significant positive correlation was observed between Top2a and the histone methyltransferase, Ezh2. Combination of the Top2 poison etoposide with the Ezh2 inhibitor GSK126 or DZNep significantly increased cell death *in vitro* in murine and human prostate cancer cell lines. Additionally, combination therapy extended time to progression and increased therapeutic efficacy *in vivo*. Overall, our studies demonstrate that patients screened for Top2a and Ezh2 expression would exhibit significant response to a combinational treatment involving low dose etoposide combined with Ezh2 inhibition. In addition, our data suggests that this combination therapeutic strategy is beneficial against aggressive PCa, and provides strong rationale for continued clinical development.

## INTRODUCTION

Prostate cancers (PCa) which progress due to gain of androgen receptor splice variants (ARv) or neuroendocrine features act independent of androgen signaling. No therapies currently exist for these lethal PCa phenotypes. Therefore, novel treatment strategies that target non-androgen related pathways that could achieve sustainable regression of disease and are urgently required. Precision medicine has emerged has a promising strategy to identify the most efficient therapy for the patient based on tumor genomics [1-3]. Identifying potential drivers of aggressive PCa and understanding their interactions will lead to novel therapeutic combinations that can be evaluated in the clinic.

Topoisomerase IIα (Top2a) is an enzyme involved in DNA replication, transcription, recombination and chromatin remodeling [4]. Given the important role of Top2a in these processes it is not surprising that Top2a has been implicated in multiple cancers [5]. Specifically, Top2a has been demonstrated to be a prognostic marker for PCa prognosis [6, 7], and is significantly up-regulated in multiple metastatic human PCa datasets [8]. The Top2 poison, etoposide in combination with estramustine was previously included by the National Comprehensive Cancer Network (NCCN) as a standard of care treatment for castrate resistant PCa with or without neuroendocrine features [9, 10]. However, current clinical data, while promising, remains to determine the most beneficial use of etoposide in patients with advanced PCa [11-14].

We identify Top2a as a significantly up-regulated transcript in metastatic tumors from a transplantable PCa mouse model of spontaneous metastasis [15]. Analysis of patient data demonstrated that increased Top2a mRNA in primary tumors selects for patients with aggressive PCa, and identified a positive correlation between Top2a and the histone methyltrasferase, Ezh2. For this reason we tested Top2 and Ezh2 combination inhibition against PCa models *in vitro* and *in vivo*, which harbor amplified AR, ARv or neuroendocrine features.

## RESULTS

### Topoisomerase IIα (Top2a) mRNA is increased in murine and human metastatic prostate cancer

**Figure 1:**
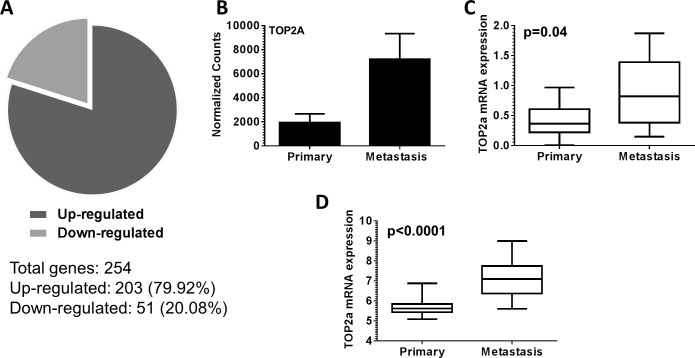
Topoisomerase IIα (Top2a) is up-regulated in murine and human metastatic prostate cancer (A) A total of 254 differentially expressed genes in metastatic versus primary disease were identified. This consisted of 203 (79.92%) genes up regulated, and 51 (20.08%) genes down regulated. (B) Normalized raw counts from RNA-seq analysis demonstrate Top2a is up regulated in murine metastatic prostate cancer. (C) Quantitative real time PCR was performed to validate increased Top2a mRNA expression in metastatic murine prostate cancer, p=0.04. (D) Analysis of Top2a mRNA expression from published human data [16] demonstrates Top2a mRNA levels are increased in human metastatic prostate cancer, p<0.0001.

To identify changes in gene expression between primary and metastatic Myc-CaP tumors (aggressive disease progression), we examined primary (n=3) and metastatic tumor tissue (n=4) collected from tumor bearing mice. Gene expression evaluation revealed a total of 254 genes were differentially expressed between metastatic tumors compared to primary tumors. Further, 203 genes (79.92%) displayed increased expression, while 51 genes (20.08%) displayed decreased expression in metastatic tumors (Figure 1A). Examination of our 254 gene signature by DAVID gene ontology (GO) analysis software revealed an enrichment of terms predominately associated with chromosome and DNA processes (supplement Figure 1a and b). From our performed DAVID analysis, we focused our attention on *topoisomerase IIα (Top2a)* for further evaluation. *Top2a* (Top2a) mRNA expression from our normalized RNA-seq counts was increased in metastatic tumors (Figure 1B). This significant increase of Top2a expression was validated by qRT-PCR from RNA samples used for RNA-seq, as well as additional independent tumor samples (p=0.04) (Figure 1C). To add clinical significance to our initial findings, we investigated the mRNA expression of Top2a in a recently published dataset of human prostate cancer from Memorial Sloan Kettering Cancer Center (MSKCC, GEO: GSE211032) [16]. In line with our mouse data, human metastatic prostate cancer exhibited significantly increased mRNA expression of Top2a compared to primary tumors (Figure 1D, p<0.0001) [16].

### Increased Top2a mRNA selects for patients with aggressive PCa

**Figure 2:**
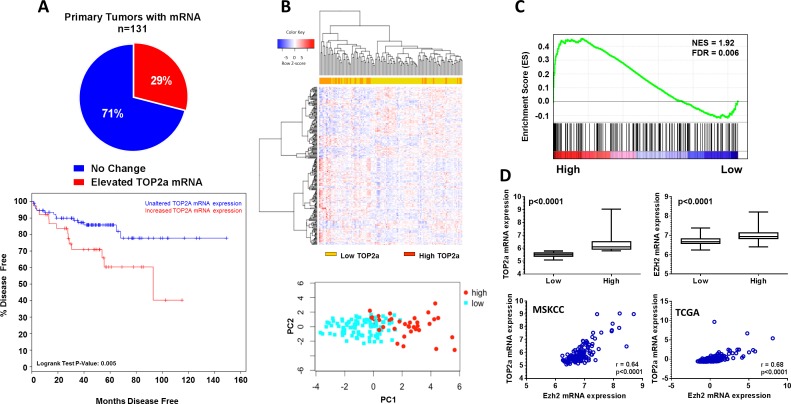
Increased Top2a expression selects for aggressive human prostate cancer and positively correlates with increased histone methlytransferase expression, Ezh2 (A) Interrogation of human primary prostate cancer samples through the cBioPortal for Cancer Genomics, show increased Top2a significantly selects for patients with aggressive prostate cancer, log rank test p=0.005. (B) Supervised hierarchical clustering and principle component analysis using the top 100 significantly altered genes demonstrate patients with low and high Top2a express unique gene signatures. (C) gene set enrichment analysis (GSEA) for oncogenic signatures using high Top2a patients expression profile show a gene set defined by up regulation as a result of increased Top2a expression (PRC2_EZH2_UP.V1_UP). (D) gene expression confirmation that expression of Top2a and Ezh2 are concurrently and significantly up regulated in human tumors with increased Top2a, p<0.0001. Spearman correlation shows significant positive correlation between Top2a and Ezh2 mRNA expression in two independent human cohorts MSKCC and TCGA, p<0.0001.

Further *in silico* analysis of primary patient tumors from the MSKCC data set (n=131) revealed that Top2a mRNA was elevated in 29% of patients (38/131 tumors), and could identify patients with aggressive disease (Figure 2A; p=0.005).

We next examined whether differences in Top2a expression in primary prostate tumor samples could identify distinct patient populations. For this, we generated differential gene signatures from patient primary tumors with high Top2a mRNA expression (Top2a high, n=38) and patient primary tumors without altered Top2a mRNA expression (Top2a low, n=93). We performed supervised hierarchical clustering and principle component analysis using the top 100 differentially expressed genes from Top2a high and Top2a low primary prostate tumors (Figure 2B). Based on this analysis we could successfully separate patients based on Top2a mRNA expression levels.

### Patients with high Top2a mRNA demonstrate positive correlation with increased Ezh2 mRNA

We performed gene set enrichment analysis (GSEA) with our Top2a high human gene signature and found enrichment of a gene signature involving the histone methlytransferase, enhancer of zeste homolog 2 (Ezh2) [17] (Figure 2C). We further confirmed association of Top2a and Ezh2 by first observing that primary human tumors with increased Top2a mRNA concurrently displayed significantly increased mRNA levels of Ezh2. Spearman correlation analysis validated further a significant positive association between levels of Top2a and Ezh2 mRNA levels in 2 independent human primary tumor datasets [16, 18, 19] (Figure 2D).

### Targeting of Top2 and Ezh2 in combination demonstrates superior anti-tumor activity *in vitro* and *in vivo*

**Figure 3:**
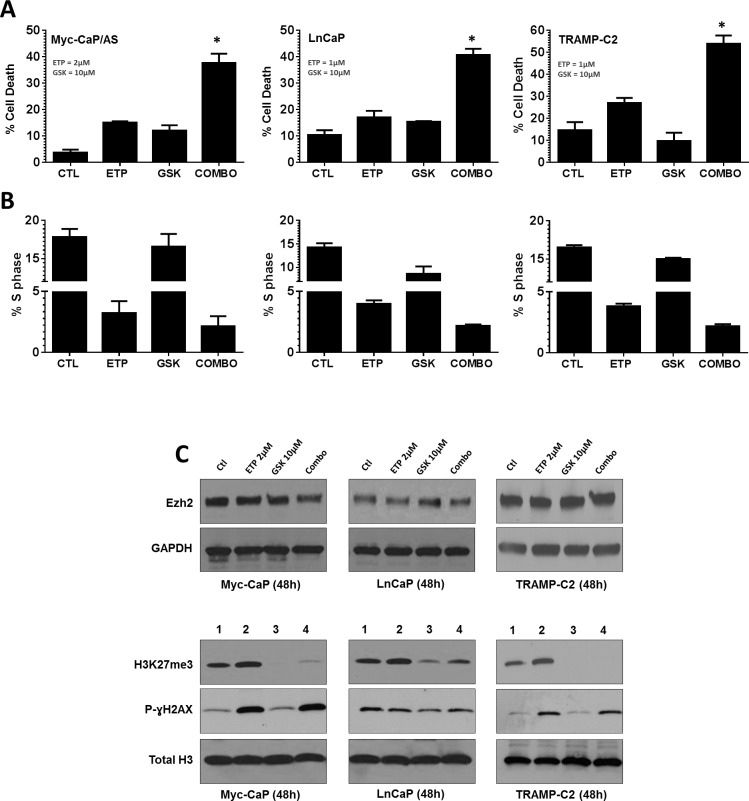
Inhibition of Top2 and Ezh2 in combination increases anti-tumor response in murine and human models of prostate cancer PCa cell lines were treated with indicated drug concentrations for 48h. (A) Cell death was assessed by incubating viable cells with propidium iodide (PI) and measuring uptake by flow cytometry, p<0.05. (B) cell cycle analysis was performed by fixing cells in 50% ethanol/PBS, before staining with PI and assessing cell cycle distribution by flow cytometry. (C) Whole cell lysates (upper panels) or histone extractions (lower panels) were generated to perform immunoblot analysis for Ezh2, H3K27me3 and p-γH2AX expression was performed 48h post treatment. GAPDH and Total Histone H3 served as loading controls.

The Top2 poison etoposide and the Ezh2 inhibitor GSK126 [20] were both tested *in vitro* for their ability to induce cell death in the murine and human PCa cell lines, Myc-CaP [21] and LnCaP [22]. Cell death in response to GSK126 in both cell lines occurred in a dose dependent manner, whereas etoposide induced cell death in both cell lines was time and dose dependent (supplement Figure 3a and b). Combination of non-cytotoxic concentrations of etoposide with GSK126 resulted in a significant increase in cell death in murine PCa cell lines Myc-CaP and TRAMP-C2 [23], and the human PCa cell line LnCaP (p<0.05). Cell cycle analysis revealed that all cell lines displayed a similar response to drug treatment. This response was demonstrated by a strong induction of cellular aneuploidy (>4N DNA content) and greatest loss of S phase within the diploid cell population (supplement Figure 2c and Figure 3B). This response within the cell cycle was primary mediated by etoposide or combination treatment. Further, both GSK126 and combination treatment resulted in loss of Ezh2 methyl-transferase activity as indicated by loss of histone H3 lysine 27 tri-methylation (H3K27me3) (Figure 3C).

Etoposide induces cytotoxic activity through interaction with Top2, forming complexes that prevent relegation of DNA; ultimately resulting in double strand DNA breaks [24]. We examined DNA double strand break (DNA-DSB) accumulation following drug treatments by p-γH2AX protein expression. As expected, etoposide increased DNA-DSB accumulation and this increase was maintained in combination treatment (Figure 3C).

**Figure 4:**
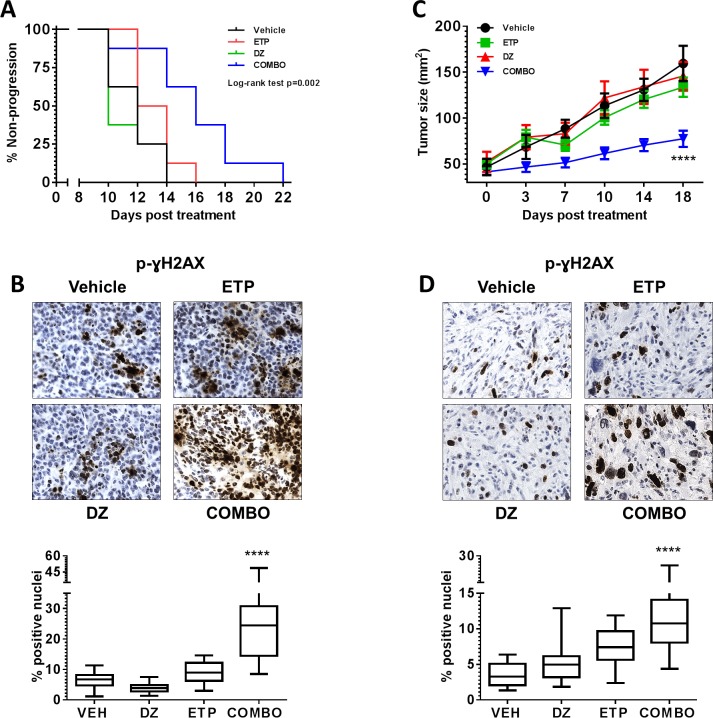
Combination inhibition of Top2 and Ezh2 increases therapeutic efficacy *in vivo* Intact male FVB or C57Bl/6 mice received 1×10^6^ Myc-CaP or TRAMP-C2 respectively by subcutaneous injection. All mice were divided into the following treatment cohorts: Vehicle (VEH: 5% DMSO/PBS, n=8), Etoposide (ETP: 10mg/kg i.p., d1-5, n=8), DZNep (DZ: 5mg/kg i.p., 2x week, n=8) or combination (n=8). (A) Combination therapy significantly delayed time to progression in mice bearing Myc-CaP tumors, p=0.002 (time to progression was considered when an individual tumor measured 2cm^2^). (C) Combination therapy significantly inhibited TRAMP-C2 tumor growth, p<0.0001. (B and D) formalin fixed tumor tissues were stained for p-γH2AX. Combination therapy significantly increased the number of positive p-γH2AX Myc-CaP and TRAMP-C2 tumor cells, p<0.0001.

Finally, the anti-tumor activity of etoposide in combination with the Ezh2 inhibitor DZNep [25] was evaluated *in vivo*. Tumor bearing mice were treated with vehicle (5% DMSO/PBS; 2x week), etoposide (10mg/kg i.p.: day 1-5), DZNep (5mg/kg i.p.: 2x week) or combination. No significant toxicity was observed in all therapy studies as shown by body weight measurement (supplement Figure 4a and 5a). Combination treatment of mice bearing Myc-CaP tumors resulted in significant delay in time to progression (p=0.002) (Figure 4A and supplement Figure 4b). Specifically, tumor bearing animals treated with vehicle had a median time to progression of 12 days. Both etoposide and DZNep did not result in significant antitumor activity as treated mice with either therapy displayed a median time to progression of 14 days. However, combination therapy extended median time to progression to 18 days. Analysis of hematoxylin & eosin (H&E) stained Myc-CaP tumor samples showed a larger accumulation of apoptotic cells within the combination treatment cohort (supplement Figure 4c). Immunohistochemical (IHC) staining for p-γH2AX further indicated dominant therapeutic efficacy of etoposide combined with DZNep resulted in significant increase in DNA-DSB (p<0.0001) (Figure 4B).

In line with our Myc-CaP tumor study, combination treatment of mice bearing TRAMP-C2 tumors significantly delayed tumor growth (p<0.0001) (Figure 4C and supplement Figure 5c). End point tumor weight analysis revealed superior combination therapy over either single treatment. Combination therapy resulted in a 38% and 57% tumor reduction in comparison to etoposide and DZNep respectively (DZNep vs. combo p=0.01; etoposide vs. combo p=0.02; supplement Figure 5b). IHC analysis of TRAMP-C2 tumors also displayed loss of H3K27me3 by DZNep and combination treatment groups, and a superior reduction in tumor proliferation by combination treatment (supplement Figure 5d). Like Myc-CaP tumors, combination treatment also resulted in a significant increase of DNA-DSB in TRAMP-C2 tumors (Figure 4D).

## DISCUSSION

Prostate cancers (PCa) that progress due to gain of androgen receptor splice variants (ARv) or neuroendocrine features act independent of androgen signaling. No therapies currently exist for these lethal PCa phenotypes. Therefore, novel treatment strategies targeting non-androgen related pathways that achieve sustainable regression of disease and are urgently required.

We have recently reported the generation of a syngeneic orthotopic transplant model of spontaneous prostate cancer metastasis [15]. This model was generated by the use of the murine Myc-CaP cell line [21]. Further, it was shown the Myc-CaP cell line expresses ARv. Myc-CaP ARv was demonstrated to be structurally similar with clinically relevant ARv, which function in the absence of ligand [26]. Because of this, we feel that this unique model currently represents an opportunity to discover new targets which can be implemented into pre-clinical therapeutic evaluation.

Our RNA-seq data highlighted increased expression *Topoisomerase IIα* (Top2a) in murine metastatic tumors, which was validated in a human PCa dataset [16]. Further, our data was consistent with previous pre-clinical and clinical data which associates increased Top2a mRNA and protein expression with more rapid disease recurrence and metastasis [6-8]. Interestingly, patients gene signature based on high Top2a expression were distinct from patients without alterations in Top2a levels. This highlights a potential mechanistic insight underlying aggressive PCa etiology.

Surprising to us, was the identification of a novel positive correlation between increased mRNA levels of Top2a and the histone methylatransferase, Ezh2. With this, we pursued the attractive approach of combination targeting of Top2 and Ezh2 inhibition. Top2 inhibitors/poisons such as etoposide have been approved by the US Food and Drug Administration for the treatment of multiple cancer cancers including PCa [4, 9, 11-14, 27]. While studies have demonstrated the potential of etoposide treatment for advanced PCa, they have yet to deliver a combination strategy that provides significant clinical benefit without associated toxicities. Also, Ezh2 is demonstrated to be deregulated in multiple cancers, including PCa [28], and has been associated with PCa progression and aggressiveness [29]. Recently, Ezh2 inhibition by compounds such as DZNep and GSK126 has proven to display *in vitro* and *in vivo* anti-tumor activity [20, 25, 30].

Combination treatment displayed strong efficacy against our murine and human PCa models both *in vitro* and *in vivo*, though combination appeared more potent in our murine PCa models, with regards to cell cycle. We believe the cell cycle responses may be enhanced in Myc-CaP and TRAMP-C2 cells compared to LnCaP cells because of more rapid doubling times of murine cell lines.

It is known that both Top2a and Ezh2 (as part of the polycomb repressive complex 2, PRC2) promote proliferation and localize at DNA replication forks [31, 32]. We demonstrate *in vitro* that increased accumulation of DNA-DSBs, aneuploidy and loss of S phase cells indicate that our observed anti-tumor activities are a result of disruption of DNA replication forks. Further, our *in vitro* data demonstrate the accumulation of DNA-DSBs was induced, as expected by etoposide, and maintained in combination treatment. While there was no difference between etoposide and combination induction of DNA-DSBs *in vitro*, our *in vivo* results showed that indeed combination treatment significantly sustained increased accumulation of DNA-DSBs over etoposide. Overall, we believe sustained interference with DNA replication are a major contributing factor to a greater cell catastrophe leading to increased cell death following combination treatment.

In summary, we demonstrate increased Top2a mRNA expression in murine and human metastatic PCa. Further, increased Top2a mRNA expression in primary human PCa samples selects for patients with more aggressive disease. We further describe a novel positive correlation between Top2a and Ezh2 mRNA expression in human PCa samples. Combination of etoposide with Ezh2 inhibition results in greater accumulation of DNA-DSB and cell death, resulting in superior anti-tumor activity and therapeutic efficacy with minimal acute toxicity *in vivo*. Our results indicate this novel combination therapeutic strategy is beneficial against aggressive PCa models expressing ARv or neuroendocrine features, and provide strong rationale for continued clinical development.

## MATERIALS AND METHODS

### Cell culture and reagents

The Myc-CaP (Myc-CaP/AS) cell line [21] was a kind gift from Dr. Charles Sawyers. Both Myc-CaP/AS and Myc-CaP/CR [33] cell lines and were cultured in DMEM medium (Gibco) supplemented with 10% fetal bovine serum and 1% penicillin/streptomycin at 37°C, 5% CO_2_. TRAMP C2 cell lines were a kind gift from Dr. Barbara Foster. TRAMP C2 cell lines were cultured in DMEM medium (Gibco) supplemented with 10% fetal bovine serum and 1% penicillin/streptomycin at 37°C, 5% CO_2_. LnCaP cell lines were purchased from ATCC, and cultured in RPMI medium (Gibco) supplemented with 10% fetal bovine serum and 1% penicillin/streptomycin at 37°C, 5% CO_2_. Primary antibodies towards Ezh2, GAPDH, H3K27me3, Total Histone H3, LC3 and p-γH2AX, activated caspase-3 were purchased from Cell Signaling. Ki-67 was purchased from Thermo Scientific. Etoposide (Sigma-Aldrich) and GSK126 (Xcess Biosciences Inc.) were maintained in DMSO at 1mM and 10mM stock concentrations respectively. Synthetic androgen (R1881) (Toronto Research Chemicals) was maintained at a 10mM stock in 100% ethanol. DZNep (Cayman Chemicals) was maintained in DMSO (10mg/ml) and diluted in PBS (1mg/ml) before use. Etoposide was obtained from the Roswell Park Cancer Institute Pharmacy Department (20mg/ml), and was diluted in PBS (2mg/ml) before use.

### RNA extraction

Freshly dissected primary and metastatic Myc-CaP/AS and Myc-CaP/CR tumor samples were immediately placed in TRIzol and homogenized (Branson Ultrasonics). Standard TRIzol/chloroform RNA extraction was then performed to isolate RNA.

### RNA-seq library preparation and sequencing

One microgram (1μg) of total RNA from Myc-CaP tumor tissue was prepared for Illumina paired-end sequencing using a Hi-Seq 2000 sequencer (Illumina). Complete descriptions for library preparation methods and sequencing data analysis are provided as supplementary online material.

Following ribosomal RNA depletion and fragmentation of total RNA (500ng), first strand cDNA is generated using reverse transcription and random primers using the TruSeq Stranded Total RNA Sample Preparation kit (Illumina, Inc.), following manufacturer's instructions. Second strand cDNA synthesis is followed by end modification and ligation of indexed sequencing adapters. The products are PCR amplified for 15 cycles, purified and validated for size (200-400bp) and quantitated using an Agilent High Sensitivity Bioanalyzer Chip and the Agilent 2100 expert software. The individual cDNA libraries are normalized to 10 nM and combined as equal molar aliquots into pools of 2-6 samples. Each pool is normalized to 10pM, loaded and clustered to individual lanes of a HiSeq Flow Cell using an Illumina cBot (TruSeq PE Cluster Kit v3), followed by 2 × 101 PE sequencing on a HiSeq2000 sequencer according to the manufacturer's recommended protocol (Illumina Inc.). The sequencing libraries were prepared with the TruSeq Small RNA kit (Illumina Inc), from 1ug total RNA. Following manufacturer's instructions, the first step involves ligation of 5′ and 3′ RNA adapters to the mature miRNAs 5′-phosphate and 3′-hydroxyl groups, respectively. Following cDNA synthesis, the cDNA is then amplified with 11-13 cycles of PCR using a universal primer and a primer containing one of 48 index sequences. The 48 different indexed tags allow pooling of libraries and multiplex sequencing. Prior to pooling, each individual sample's amplified cDNA construct is visualized on a DNA-HS Bioanalyzer DNA chip (Agilent Technologies) for mature miRNA and other small RNA products (140-150bp). Successful constructs are purified using a Pippen prep (Sage Inc.), using 125 – 160 bp product size settings with separation on a 3% agarose gel. The purified samples are validated for size, purity and concentration using a DNA-HS Bioanalyzer chip. Validated libraries are pooled equal molar in a final concentration of 10nM in Tris-HCI 10 mM, pH 8.5, before 50 cycle sequencing on a MiSeq (Illumina, Inc.).

### RNA-deep sequencing analysis

All analysis was performed by the Department of Bioinformatics and Statistics at Roswell Park Cancer Institute. Reads were mapped to the latest mouse reference genome (mm10) using Bowtie [34]. From the Bowtie results, reads that matched a single unique location in the genome were identified, allowing up to two mismatches. The number of reads aligning to each gene was calculated. Between-sample normalization was performed using the Trimmed Mean of M-values normalization method [35], which is specifically designed for RNA-seq data. Differentially expressed genes were identified using DESeq [36], a variance-analysis package developed to infer the statically significant difference in RNA-seq data. Multiple testing corrections were corrected. GEO accession number, GSE64771, for RNA seq.

*Gene Ontology (GO) David Analysis*: GO enrichment analysis was performed with DAVID functional classification tool. The generated DEG file for metastatic genes was uploaded to the web based bioinformatics tool DAVID (http://david.abcc.ncifcrf.gov/tools.jsp) [37]. Cutoff for genes of interest in the DEG file was a p-value of <0.05.

*Gene Set Enrichment Analysis*: The DEG genes for the metastatic tumors were uploaded into the JAVA based GSEA (http://www.broadinstitute.org/gsea) [38] tool and the oncogenic signatures dataset were selected. Gene lists from our RNA-seq as well as from the publically available clinical dataset by Taylor *et al* [16] were uploaded and analyzed.

*In silico analysis of human prostate cancer data sets*: The human prostate cancer dataset was utilized through the cBioPortal for Cancer Genomics [18, 19]. Kaplan-Meier survival plot was generated by CBioPortal and log rank statistical test applied. Gene signatures were generated using Biobase [39] and Lima [40] for primary tumors with increased Top2a expression (Top2a high) and primary tumors samples without increased Top2a expression (Top2a low). Supervised hierarchical clustering and principle component analysis was performed using the top 100 genes that were significantly changed between either human primary prostate tumors with or without increased Top2a expression (p<0.05, fold change >1.5) using gplots [41]. Gene set enrichment analysis (GSEA) for oncogenic signatures of human high Top2a expression profile was performed using JAVA based GSEA tool [38]. Spearman correlation analysis was used to validate a significant positive correlation between Top2a and Ezh2 mRNA expression in two independent human cohorts (MSKCC [16] and TCGA [18, 19]).

### Quantitative real-time PCR

Synthesis of cDNA was performed according to iScript cDNA Synthesis Kit (Bio-Rad Laboratories, Hercules, USA). 1μg of RNA was added to a master mix containing nuclease free H_2_O, and reagents (5X iScript reaction mix + iScript reverse transcriptase) from the kit in a total volume of 20μl. cDNA was diluted 1:4 prior to qRT-PCR. PCR primers were designed with NCBI's primer blast tool (http://www.ncbi.nlm.nih.gov/tools/primer-blast/), with a melting temperature 57-63°C and a resulting product size of 75-200bp. Primers were obtained from Integrated DNA technologies (Coralville, USA). Primer sequences were *Top2a* (F: AGG ATT CCG CAG TTA CGT GG, R: CAT GTC TGC CGC CCT TAG AA), and *GAPDH* (F: GTCTTCACCACCATGGAGAAG, R: CAAAGTTGTCATGGATGACCTTGG). Each PCR reaction was carried out in technical triplicates in a 10μl volume utilizing SYBR Green Master Mix (Bio-Rad Laboratories, Hercules, USA). GAPDH was used a control gene. The resulting Ct-values for each gene were normalized to the expression values of GAPDH. The fold change of metastatic tumor samples was then calculated relative to that of primary tumor samples.

### *In vitro* cell death assays

Cells were incubated in the presence of either in single or combination dose of Etoposide and GSK126 for 24 or 48 hours respectively. Viability of cells was measured by trypan blue exclusion assay. Cells were mixed at a 1:1 (v/v) ratio with trypan blue (0.4% in PBS) (Corning Cellgro). Cell death was then determined by counting a total of 100-cells in a haemocytometer using a light microscope.

### Cell cycle analysis

Cells were seeded into 6-well plates (BD Bioscience), left to adhere, and treated as indicated. Following treatments, adherent and non-adherent cells were collected and washed in 1x PBS, and fixed in 50% ethanol at 4ºC overnight. Cells were stained with propidium iodide solution containing RNase A (Sigma) for 15 minutes at 37ºC. DNA content analysis was performed by using a FACS caliber cytometer.

### Western Blot Analysis

*Whole Cell Lysate Preparation (WCL):* Cells were harvested and lysed with RIPA buffer (Sigma Aldrich, USA) + 1X P-STOP + 1X PIC (Roche) for 30 minutes on ice. Eppendorf tubes were vortexed every ten minutes for 10 seconds. After cell lysis tubes were centrifuged at 13,000 rpm for 15 minutes at 4°C. Supernatant of each tube was collected and transferred to a new tube.

*Histone Extraction:* Histone extractions were performed using the Epigentek (EpiQuik Total Histone extraction kit OP-0006) histone extraction kit.

Protein concentrations of whole cell lysates (WCL) and histone extractions were measured by the bradford protein assay (Bio-Rad laboratories). Protein lysates (50μg WCL, 5μg Histone Extraction) where separation using 4-15% by SDS-PAGE gels (Bio-Rad). The proteins were transferred from the SDS-PAGE gel onto nitrocellulose membrane (0.2 μm) (Bio-Rad, Hercules, CA) via the semi-dry method (Bio-Rad, Hercules, CA) for 35 minutes at 15V. Membranes were blocked in either 5% skim milk or BSA in 0.1% tween-PBS (tPBS) for 1-hour at RT. Membranes were washed briefly 3x with tPBS prior to primary antibody incubation at 4°C over night. Membranes were then washed 3×10 minutes before the addition of secondary horseradish peroxidase (HRP)-conjugated antibodies (Bio-Rad, Hercules, CA) diluted in tPBS. After incubation at RT for 1-hour with agitation the membranes were washed 3×10 minutes in tPBS. The immunoreactive bands were visualized by enhanced chemiluminescence with ECL detection reagents (GE Healthcare Life Sciences, UK). The blots were exposed to Bio film for 1 second-10 minutes. The films were then developed in a Kodak film developer. To estimate molecular weight of bands a pre-stained protein ladder was used (Bio-Rad, Hercules, CA).

### *In vivo* animal studies

The Institute Animal Care and Use Committee at Roswell Park Cancer Institute approved all mouse protocols used in this study. One million (1×10^6^) Myc-CaP/AS cells, or five million (5×10^6^) TRAMP C2 cells were subcutaneously injected into intact FVB and C57-Bl/6 male mice respectively. Treatment was initiated when tumor size reached ~40mm^2^, and mice were randomized into four treatment groups: (1) Vehicle (5% DMSO/PBS, 2x week, intraperitoneal (i.p.) injection), (2) Etoposide (10mg/kg, d1-5, i.p.), (3) DZNep (2mg/kg, 2x week, i.p.), (4) combination. Mice were weighed weekly to monitor for toxicity and tumor growth was assessed by serial caliper measurements twice weekly.

### Immunohistochemistry

Mice were sacrificed by CO_2_ asphyxiation at defined time points. Tumor was fixed in 10% buffered formalin overnight followed by an additional 24 hours in 70% ethanol. For antigen retrieval, slides (4μM) were boiled for 10 minutes in 10mM sodium citrate (pH 6) solution for all antibodies. ImmPRESS detection sytem (Vector Labs) was used for detection of all primary antibodies. Staining was visualized using 3,3′-Diaminobenzidine (DAB) (Sigma). Slides were counterstained with hematoxylin. Quantitation of IHC staining representative images (4-6) was obtained using a Zeiss light microscope (Zeiss). Staining intensity was scored by Aperio ImageScope (v11.1.2.760).

### Statistical analysis

Data are displayed as mean ±SEM. Differences were determined using two-tailed unpaired t-tests and two-way ANOVA, using GraphPad Prism software. P values less than 0.05 were assigned statistically significant.

## SUPPLEMENTARY MATERIAL AND FIGURES


