# Supplementary material for: Top2a identifies and provides epigenetic rationale for novel combination therapeutic strategies for aggressive prostate cancer
Source: Oncotarget. 2014 Dec 26;6(5):3136–46. doi: 10.18632/oncotarget.3077 (PMC4413643; doi:10.18632/oncotarget.3077)
Supplement: Supplementary file 1 [file oncotarget-06-3136-s001.pdf]

Top2a identifies and provides epigenetic rationale for novel combination therapeutic strategies for aggressive prostate cancer

Supplementary Material

A

Category	Term	Gene Count	FDR
Biological Process	GO:0051301~cell division	31	6.93E-17
Biological Process	GO:0007059~chromosome segregation	12	4.36E-07
Biological Process	GO:0006323~DNA packaging	11	6.60E-04
Biological Process	GO:0051276~chromosome organization	17	0.0562
Biological Process	GO:0006259~DNA metabolic process	13	8.4573
Cellular Component	GO:0005694~chromosome	30	5.44E-13
Cellular Component	GO:0000793~condensed chromosome	18	7.14E-12

B

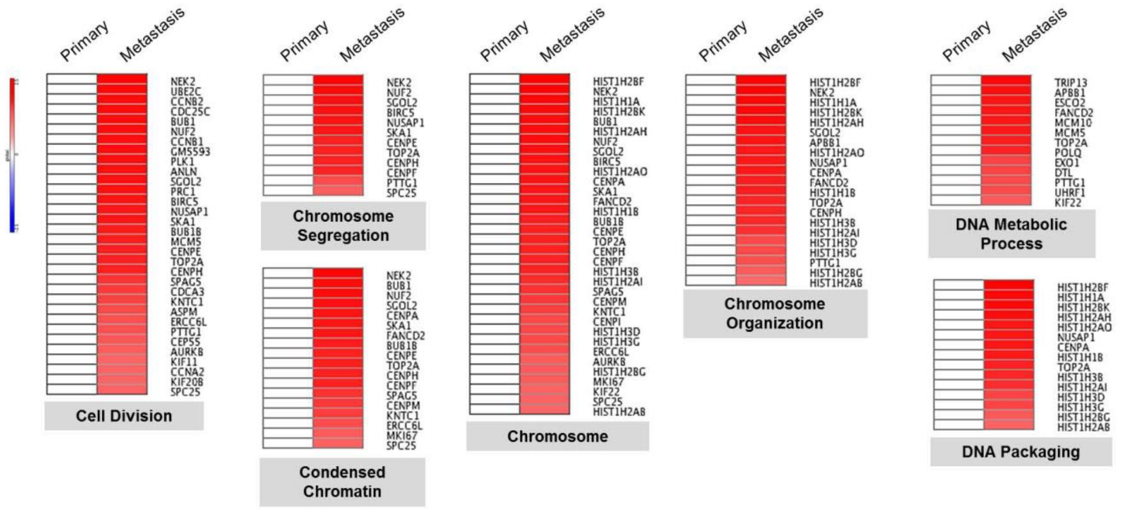

Supplement Figure 1: RNA-seq analysis of Myc-CaP primary and metastatic tumors. (A) Gene Ontology (GO) enrichment analysis was performed with DAVID. (B) Heat maps of DAVID GO categories from (A).

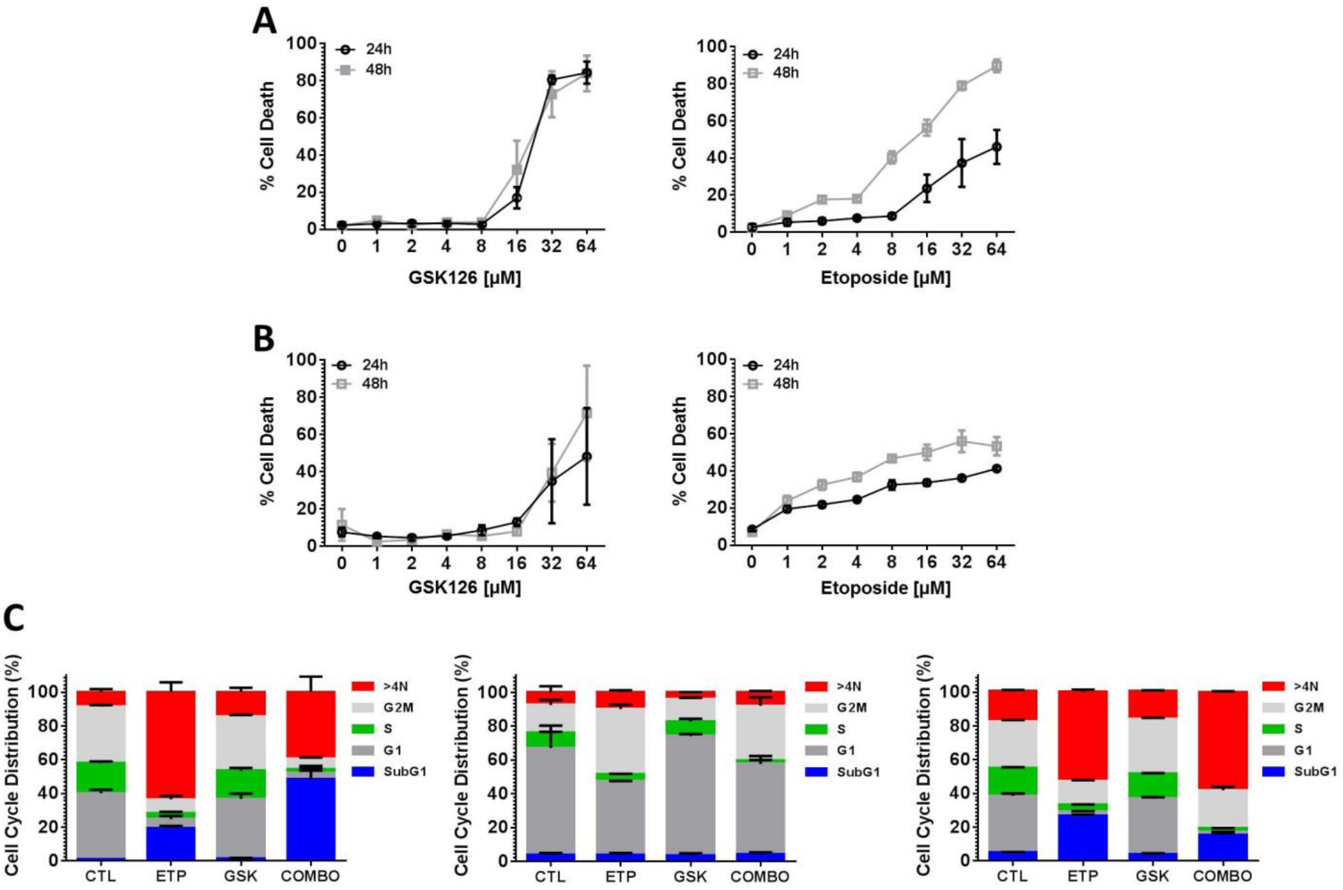

Supplement Figure 2: PCa cell line response *in vitro* to inhibition of TOP2 and EZH2. (A) murine Myc-CaP and (B) human LnCaP PCa cell lines were treated with the EZH2 inhibitor; GSK126 (GSK) or the TOP2 inhibitor; Etoposide (ETP) as indicated. Cell death was assessed by uptake of propidium iodide (PI), quantitated by flow cytometry. (C) Cell cycle analysis of Myc-CaP, LnCaP and TRAMP-C2 cell lines respectively. Cells were treated as indicated for 48h then fixed in 50°/o ethanol/PBS overnight. Cells were stained with PI and analyzed by flow cytometry.

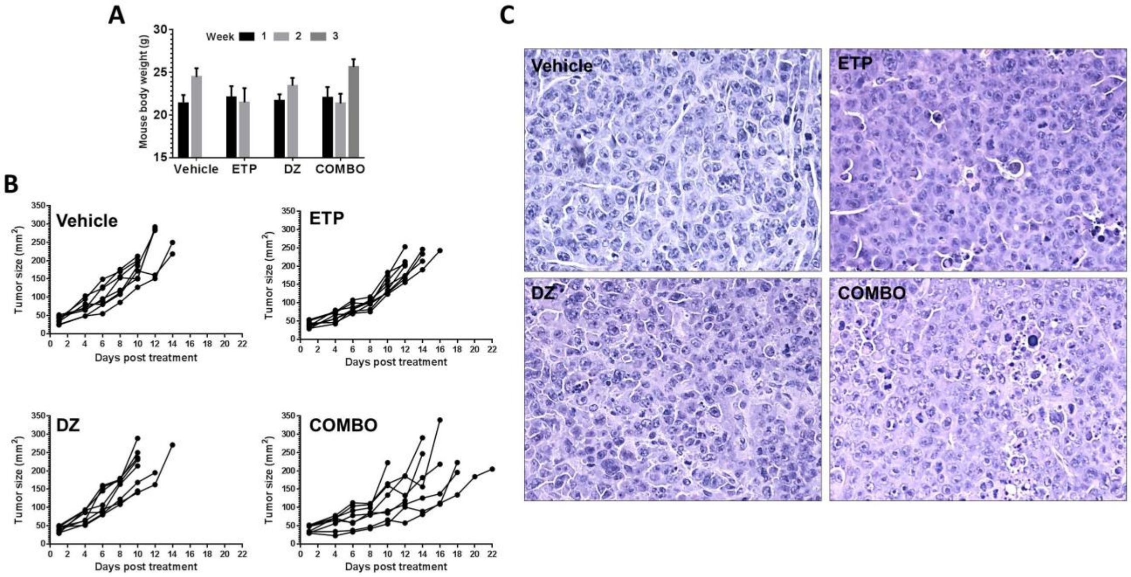

Supplement Figure 3: Combination inhibition of TOP2 and EZH2 increase therapeutic efficacy *in vivo*. (A) Weekly body weights of tumor bearing animals indicates no toxicity was induced within treatment groups. (B) Individual tumor measurements from figure 4A. (C) Example micrographs of FFPE Myc-CaP tumor samples stained with Hematoxylin and Eosin (H&E). Magnification x40.

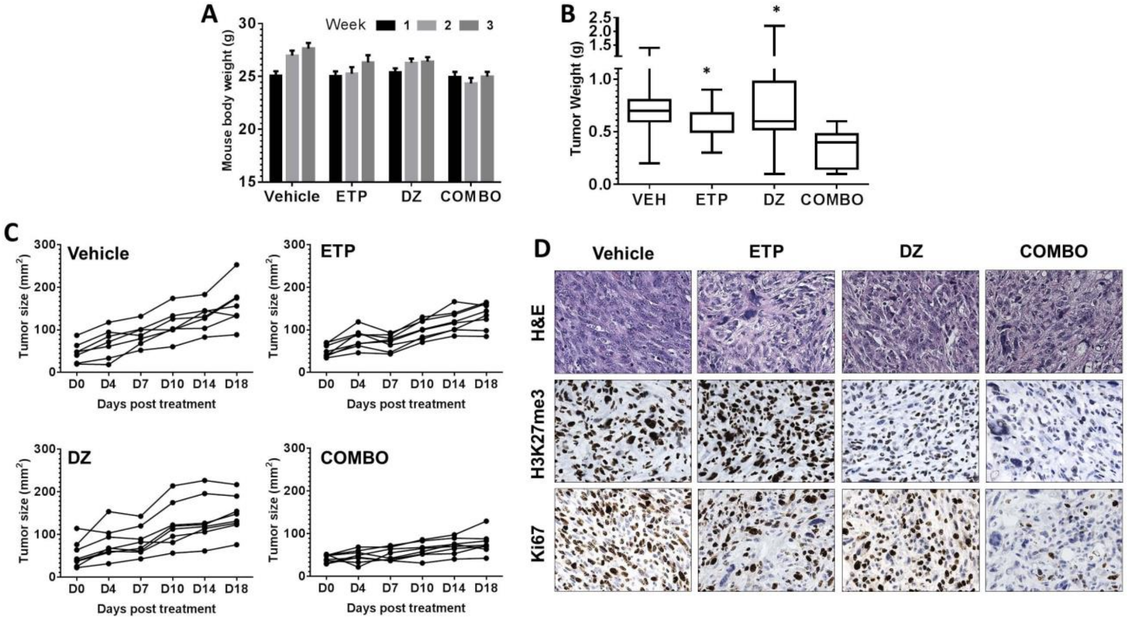

Supplement Figure 4: Combination inhibition of TOP2 and EZH2 increase therapeutic efficacy *in vivo.* (A} Weekly body weights of tumor bearing animals indicates no toxicity was induced within treatment groups. (B) Endpoint TRAMP-C2 tumor weights (DZ vs. COMBO *p=0.01; ETP vs. COMBO *p=0.02). (C) Individual TRAMP-C2 tumor measurements from figure 4C. (C) Example micrographs of FFPE TRAMP-C2 tumors stained with H&E; Immunohistochemistry (IHC) for H3K27me3, Ki67. Magnification x40.
